# Systematic review of the therapeutic use of Schwann cells in the repair of peripheral nerve injuries: Advancements from animal studies to clinical trials

**DOI:** 10.3389/fncel.2022.929593

**Published:** 2022-07-29

**Authors:** Frederic A. Vallejo, Anthony Diaz, Emily L. Errante, Taylor Smartz, Aisha Khan, Risset Silvera, Adriana E. Brooks, Yee-Shuan Lee, Stephen Shelby Burks, Allan D. Levi

**Affiliations:** ^1^Department of Neurosurgery, University of Miami Miller School of Medicine, Miami, FL, United States; ^2^Department of Neurosurgery, University of Connecticut, Farmington, CT, United States; ^3^Miami Project to Cure Paralysis, University of Miami Miller School of Medicine, Miami, FL, United States

**Keywords:** Schwann cells (SCs), peripheral nerve, nerve regeneration, Schwann cell transplantation, nerve repair

## Abstract

**Objective:**

To systematically evaluate the literature on the therapeutic use of Schwann cells (SC) in the repair of peripheral nerve injuries.

**Methods:**

The Cochrane Library and PubMed databases were searched using terms [(“peripheral nerve injury” AND “Schwann cell” AND “regeneration”) OR (“peripheral nerve injuries”)]. Studies published from 2008 to 2022 were eligible for inclusion in the present study. Only studies presenting data from *in-vivo* investigations utilizing SCs in the repair of peripheral nerve injuries qualified for review. Studies attempting repair of a gap of ≥10 mm were included. Lastly, studies needed to have some measure of quantifiable regenerative outcome data such as histomorphometry, immunohistochemical, electrophysiology, or other functional outcomes.

**Results:**

A search of the PubMed and Cochrane databases revealed 328 studies. After screening using the abstracts and methods, 17 studies were found to meet our inclusion criteria. Good SC adherence and survival in conduit tubes across various studies was observed. Improvement in morphological and functional outcomes with the use of SCs in long gap peripheral nerve injuries was observed in nearly all studies.

**Conclusion:**

Based on contemporary literature, SCs have demonstrated clear potential in the repair of peripheral nerve injury in animal studies. It has yet to be determined which nerve conduit or graft will prove superior for delivery and retention of SCs for nerve regeneration. Recent developments in isolation and culturing techniques will enable further translational utilization of SCs in future clinical trials.

## Introduction

It has been estimated that 2–3% of all patients admitted to Level 1 Trauma centers present with a peripheral nerve injury, and injury to peripheral nerves has been observed in 1.64% of patients who experience trauma to the upper or lower extremities (Noble et al., 1998; Taylor et al., 2008). A recent study revealed an overall peripheral nerve injury incidence rate of 36.9 per 1,000,000 person years in sport, exercise, and recreational injuries presenting to the emergency room from 2009 to 2018 (Li et al., 2021). These injuries can be devastating to patients, resulting in potential losses of both sensory and motor function in the affected distribution, and are associated with significant costs to health system infrastructure (Karsy et al., 2019; Bergmeister et al., 2020).

Short-segmental gap peripheral nerve injuries can be readily repaired by end-to-end suturing, autologous nerve transplantation, processed nerve allografting, and conduit implementation. Long-gap peripheral nerve injuries, however, have posed significant challenges to surgeons and require novel approaches to effectively restore nerve patency and enhance post-operative return of function. The “critical” gap length for long-gap injuries has been established as 3 cm in humans and 10–15 mm in rats, the model most commonly utilized in peripheral nerve repair studies (Lundborg et al., 1982; Francel et al., 1997; Kaplan et al., 2015). Longer nerve defects (>5 cm) have been shown to independently correlate with significantly worse outcomes for patients (Roganovic et al., 2005). Autologous sural nerve transplantation, long-considered the gold standard for repair of these injuries, poses multiple limitations. Sural nerve harvesting can increase patient morbidity by necessitating a separate incision and leading to potential injury due to sensory loss. Limitations in nerve supply for autologous transplantation in repair of nerves with large cross-sectional areas, such as in sciatic and femoral nerve transections, may lead to suboptimal outcomes (Burks et al., 2021). Nerve allografts and conduits have been investigated as alternatives to harvesting nerves for autologous grafting (Angius et al., 2012). Allografted nerves containing living cells require treatment with immunosuppressant agents to mitigate rejection, and though processed nerve grafts and acellular conduits alone have been implemented successfully in short-gap nerve repair, their use for long-gap repair remains controversial with mixed results (Midha et al., 1993; Den Dunnen et al., 1996; Isaacs and Browne, 2014; Muheremu and Ao, 2015; Leckenby et al., 2020; Safa et al., 2020).

In an effort to overcome some of these difficulties, researchers have explored the use of Schwann cells (SCs) to repair peripheral nerve injuries (Mosahebi et al., 2002; Hood et al., 2009; Han et al., 2019). In the peripheral nervous system, SCs myelinate axons and are the primary source of structure and support. These cells secrete neurotrophic factors into their micro-environments that promote axonal regeneration and nerve fiber extension after peripheral nerve injury. The regenerative effect conferred by SCs is not limited to the PNS and has been observed in the central nervous system as well (Bachelin et al., 2005; Kocsis and Waxman, 2007). As SCs have exhibited robust capabilities in facilitating axonal repair and regeneration, they carry great potential as a candidate for transplant therapy in long-segmental peripheral nerve injuries.

Harvesting, purifying, and expanding populations of SCs for autologous transplant has proved to be exceedingly difficult until recent years. The purpose of this systematic review is to detail the current role of SC implementation in peripheral nerve repair, and to outline current limitations as well as future clinical applications and directions for this technology.

## Methodology

### Literature search strategy

We searched PubMed and Cochrane databases to find articles published on this review topic. The following terms were utilized: [(“peripheral nerve injury” AND “Schwann cell” AND “regeneration”) OR (“peripheral nerve injuries”)]. Our search was limited to studies published in English and was updated until March 27, 2022. Upon removal of duplicates, two researchers screened each study independently, reviewing their abstracts and methodology to ascertain whether each would be pertinent to this study. Finally, included studies were reviewed in their entirety and discussed herein. See PRISMA checklist.

### Inclusion criteria

Studies published in English from 2008 to 2022 were eligible for inclusion in the present study. Additionally, to be included, it was necessary for studies to have available abstracts for initial screening purposes. Studies were required to present data from *in-vivo* studies utilizing Schwann cells in the repair of peripheral nerve injuries with a gap of ≥10 mm. Lastly, studies needed to have some measure of quantifiable regenerative outcome data such as histomorphometry, immunohistochemistry, electrophysiology, or other functional outcomes reported.

### Exclusion criteria

Studies published in languages other than English as well as those published prior to 2008 were excluded from the present study. Studies which did not include abstracts were excluded. Review articles, opinion pieces, and studies which presented purely *in-vitro* data were excluded. Studies investigating lesions to the central nervous system such as spinal cord injuries were excluded. Articles in which stem-cells were utilized and differentiated to Schwann cell phenotypes and those that did not report quantifiable outcomes were excluded.

## Results

**Figure 1:**
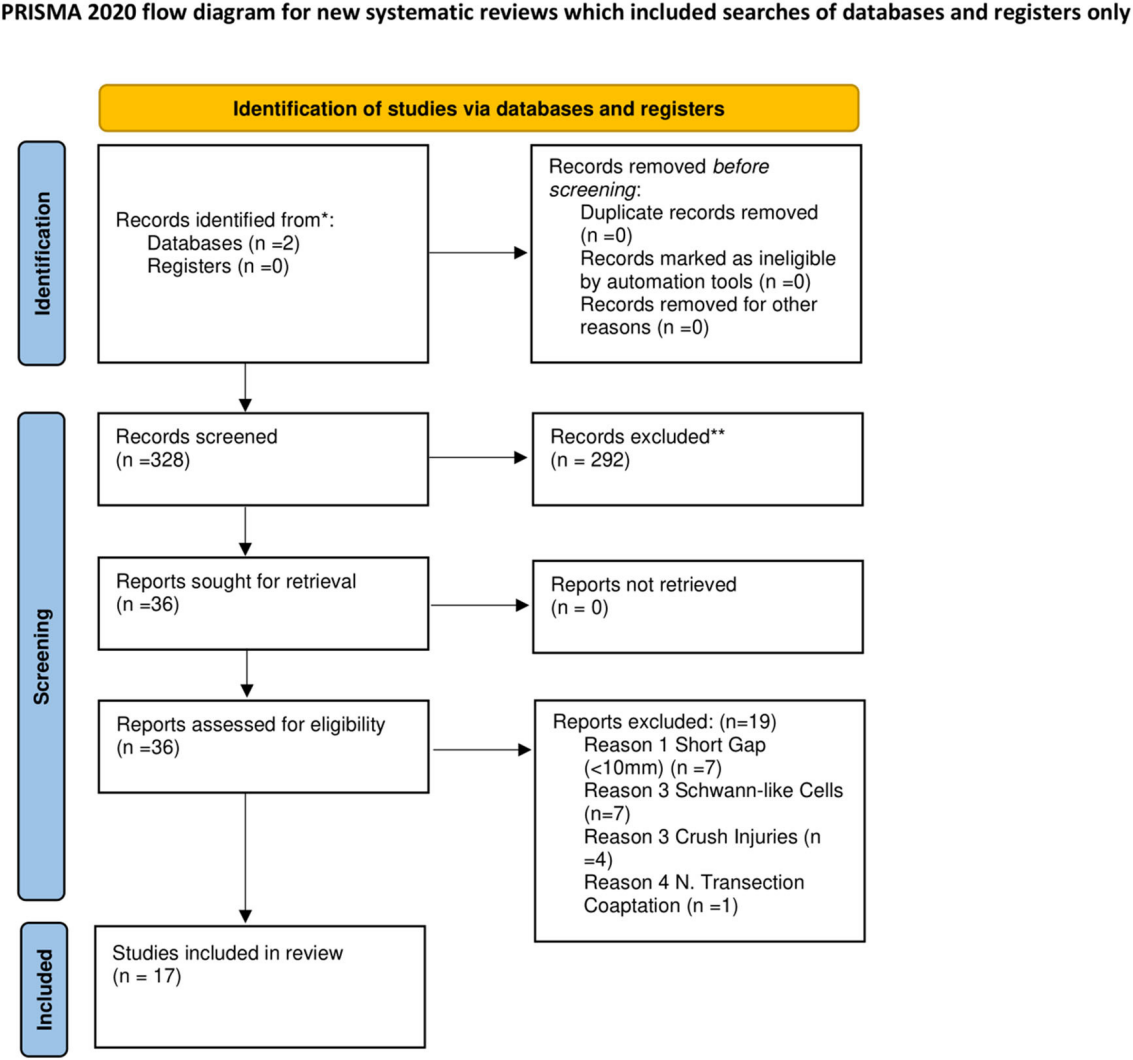
Flowchart describing literature search and study selection. ^*^Consider, if feasible to do so, reporting the number of records identified from each database or register searched (rather than the total number across all databases/registers). ^**^If automation tools were used, indicate how many records were excluded by a human and how many were excluded by automation tools. From Page et al. (2021).

A preliminary query of the PubMed and Cochrane databases revealed 328 studies from 2008 to 2022 (Figure 1). After an initial screening using the abstracts and methods, 17 studies were found to meet our inclusion criteria.

These 17 studies in which Schwann cells were used in transplantation are reviewed in Table 1. Studies on SC transplantation spanned from 2008 to 2021. Five studies utilized acellular nerve allografts, four used varying iterations of chitosan-based conduits, two studies used PCL based conduits, two used collagen-based conduits, one study used a silicone conduit, one used a cellulose conduit, one study investigated a conduit made from electro-spun Perfluorotributylamine (PFTBA), and the remaining study utilized two nanocomposite-based conduits made of silk fibroin. Thirteen studies reported the concentration of SCs loaded into the nerve conduits. Studies ranged from 4 to 52 weeks with a mean study period of ~14 weeks.

**Table 1:** Autologous Schwann cell transplantation for long-gap peripheral nerve injury.

**References**	**Animal model**	**Nerve gap**	**Controls used**	**Experimental groups**	**Cell type**	**Number of transplanted cells**	**Study period**	**Conduit used**
Aszmann et al., 2008	Sprague-Dawley and Lewis Rats	30 mm	homologous matrix only withoutSchwann cell, autograft	Study groups were (1) SD acellular autograft covered with SCs from proximal neuroma (2) Lewis rats with acellular homograft covered with SCs from proximal neuroma	Autologous SCs		12 weeks	Acellular auto- or homograft
Chang, 2009	Sprague-Dawley Rats	15 mm	Isograft	Conduit filled with (1) Genipin non-crosslink (2) low crosslink (3) medium crosslink (4) high crosslink	Autologous SCs	3 ×10^5^ cells/mL (9 ×103 cells)	8 weeks	Polycaprolactone (PCL), genipin-crosslinked gelatin
Sun et al., 2009	Wistar Rats	10 mm	Autograft	Conduit filled with (1) SCs (2) Acellular	Autologous SCs	2 ×10^6^ cells in 100 μl	12 weeks	Acellular nerve allograft
McGrath et al., 2010	Fischer Rats	10 mm	Autograft	Conduit filled with (1) BD™ PuraMatrix™ peptide (BD) hydrogel (2) Conduit filled with BD™ PuraMatrix™ peptide (BD) hydrogel plus SCs (3) Conduit filled with alginate/fibronectin hydrogel (4) Conduit filled with alginate/fibronectin hydrogel plus SCs	Autologous SCs		16 weeks	Cellulose conduit
Berrocal et al., 2013	Fischer and Lewis Rats for reversed autograft	15 mm	Autograft	Conduit filled with (1) GFP negative SCs (2) GFP positive SCs (3) serum only	Autologous SCs	2 ×10^5^ cells/μl	16 weeks	NeuraGen™
Jesuraj et al., 2014		14 mm	Isograft, cold preserved acellular nerve graft with no SCs	Conduit filled with (1) SCs derived from sciatic nerve (2) SCs derived from femoral motor (3) SCs derived from femoral sensory	Autologous SCs	10^6^ cells	7 weeks	Cold preserved acellular nerve graft
Hoben et al., 2015	Lewis Rats	20 mm	Isograft	(1) isograft (2) ANA (3) ANA-SCs (4) ANA-VEGF	Autologous SCs	10^6^ cells	10 weeks	Acellular nerve allograft
Wang et al., 2017	Nerve donors were ICR mice and graft recipients were C57BL/6 mice	10 mm	Acellular nerve allograft alone	Study groups (1) SF conduit plus SCs (2) KLF7-SCs	Allogenic SCs	2 ×10^7^ SCs in 100 μl	4 weeks	Acellular nerve allograft
Das et al., 2017	Sprague-Dawley Rats	10 mm	Sciatic nerve left untreated	Study Groups (1) Silk fibroin conduit (2) Silk fibroin conduit plus SCs (3) PASF conduit (4) PASF conduit plus SCs	Rat Schwann cell line (SCTM41)	1 ×10^5^ SCTM41 cells (rat Schwann cells)	52 weeks	Silk Fibroin (SF) or Polyaniline-silk (PASF) nanocomposite-based
Liu et al., 2017	Sprague-Dawley Rats	15 mm	Autograft	Study Groups (1) magnetic scaffold (2) magnetic scaffold under MF exposure (3) SC-loaded magnetic scaffold (4) SC-loaded magnetic scaffold under MF exposure	Autologous SCs	-	12 weeks	Magnetic nanocomposite scaffold, chitosan-glycerophosphate polymer
Ma et al., 2018	Sprague-Dawley Rats	15 mm	Autograft	Conduit filled with (1) SC and fibrin hydrogel (2) PFTBA alone without SCs (3) PFTBA plus SCs	Autologous SCs	2 ×10^6^ GFP-expressing SCs in 10 ml fibrinogen solution	12 weeks	Chitosan-collagen
Gonzalez-Perez et al., 2018	Wistar Hannover rats (Janvier)	15 mm	Acellular fibronectin enriched conduit or laminin enriched conduit	Conduit filled with (1) collagen-fibronectin 20% plus MSCs (2) collagen-fibronectin 20% plus SCs (3) collagen-laminin 20% plus MSCs (4) collagen-laminin 20% plus SCs	Allogenic SCs	7.5 ×10^5^ cells. The pellet was resuspended in 1 ml	16 weeks	Chitosan
Huang et al., 2019	Sprague-Dawley Rats	10 mm	Conduit filled with Matrigel and SCs	Conduit filled with (1) c-jun transfected SCs continuously treated with Dox for 12 wk (2) c-jun transfected SCs treated with no Dox for the entire experimental period (Dox−12) (3) c-jun transfected SCs treated with Dox for 3 wk followed by removal of Dox for 9 wk (Dox+3/−9)	Autologous SCs	SCs and matrigel (2 ×10^7^ cells/ml)	12 weeks	Poly (ε-caprolactone) (PCL)
Ma et al., 2020	Sprague-Dawley Rats	17 mm	Autograft	Conduit filled with (1) SCs without PFTBA injected into (fibers + gel) (2) SCs without PFTBA injected into (PFTBA fibers + gel) (3)SC-gel mixtures with PFTBA injected into (PFTBA-gel) (4) SC-gel with PFTBA injected into (PFTBA fibers + PFTBA-gel)	Autologous SCs	1 ×10^6^ SCs	12 weeks	Perfluorotributylamine
Burks et al., 2021	Fischer Rats	13 mm	Autograft, NeuraGen™ filled with Serum only	Conduit filled with green fluorescent protein (GFP)–labeled SCs	Autologous SCs	1 ×10^5^ cells/μl	16 weeks	NeuraGen™ 3D
Muangsanit et al., 2021	Wistar Rats	10 mm		Conduit filled with (1) SCs (2) HUVEC (3) SC-HUVEC combination	Rat Schwann cell line SCL4.1/F7	100 μl of cell suspension (culture medium containing 0.5–4.0 ×10^6^ cells/ml)	4 weeks	Silicone tube collagen gel
Yao et al., 2021	Sprague-Dawley Rats	10 mm	Conduit filled with PBS	Conduit filled with (1) Lentivirus control SCs (2) Lv-loc680254 SCs	Autologous SCs	-	12 weeks	Chitosan

### SC adherence and survival in conduit tubes

SCs were observed to have good adherence and displayed a homogenous distribution upon implantation in conduit tubes (Sun et al., 2009; McGrath et al., 2010; Berrocal et al., 2013; Das et al., 2017; Huang et al., 2019; Burks et al., 2021; Muangsanit et al., 2021). SCs also demonstrated good survival. In Berrocal et al. (2013), GFP fluorescence of autologous SCs was detected up to 16 weeks after implantation. Optimization in SC survival was observed with use of a perfluorotributylamine (PFTBA) fibrin hydrogel, an oxygen carrier, within a chitosan conduit in Ma et al. (2018). They observed a significantly greater survival of GFP labeled SCs within the PFTBA conduit up to 28 days after implantation as compared to the conduit with SCs but without the hydrogel. In Ma et al. (2020), the authors demonstrated further enhancement of SC survival in a conduit designed with a core-shell structure containing encapsulated PFTBA. The new scaffold coupled with the PFTBA hydrogel allowed for greater oxygen carrying capacity and increased SC survival during the initial hypoxic phase of nerve regeneration. Similarly, Liu et al. (2017) demonstrated improvement in SC viability and distribution in a magnetically responsive scaffold upon exposure to a magnetic field.

### Improvement in myelinated axons

Nerve regeneration was assessed through various morphologic measurements, including number of myelinated axons, axonal area/diameter, and myelin thickness. Although significant interstudy differences exist regarding the graft employed for repair, gap length, and time point for measurements, there is near complete consensus among animal studies that supplementing nerve grafts with Schwann cells in the repair of critical nerve gap defects enhances nerve regeneration.

There were 15 studies (Aszmann et al., 2008; Sun et al., 2009; McGrath et al., 2010; Berrocal et al., 2013; Jesuraj et al., 2014; Hoben et al., 2015; Liu et al., 2017; Wang et al., 2017; Gonzalez-Perez et al., 2018; Ma et al., 2018, 2020; Huang et al., 2019; Burks et al., 2021; Yao et al., 2021) that evaluated the number of myelinated axons within the graft through light microscopy and/or through immunostaining. Among those, 14 studies demonstrated significant improvement in myelinated axon counts in the graft with SCs as compared to the graft alone (Aszmann et al., 2008; Sun et al., 2009; McGrath et al., 2010; Berrocal et al., 2013; Jesuraj et al., 2014; Hoben et al., 2015; Liu et al., 2017; Wang et al., 2017; Gonzalez-Perez et al., 2018; Ma et al., 2018, 2020; Huang et al., 2019; Burks et al., 2021; Yao et al., 2021). The presence of regenerated myelinated axons in the middle of a 10-mm gap were observed as early as 10–14 days after surgery (Huang et al., 2019; Yao et al., 2021) and in the distal end of a 10-mm gap as early as 3–4 weeks (McGrath et al., 2010; Liu et al., 2017; Huang et al., 2019). Yao et al. (2021) observed that in a 10-mm gap repaired with a chitosan scaffold and injected with either native SCs or SCs transfected with a long coding RNA (loc680254) shown to increase SC proliferation, regenerated axons grew up to 5–6 mm past the proximal stump as compared to 2 mm for the empty scaffold at 10 days after surgery. In McGrath et al. (2010), regenerating axons were observed to reach as far 15.88 mm from the proximal stump in a 10-mm gap repaired with a membrane conduit filled with a BD™ PuraMatrix™ peptide (BD) hydrogel and SCs. Axons in the BD hydrogel group without SCs reached a mean distance of 8.56 mm. Hoben et al. (2015) demonstrated no significant difference in the number of total nerve fibers regenerated between isograft and acellular nerve graft loaded with SCs at 10 weeks.

In nearly all studies that employed a reversed autograft group as a positive control (McGrath et al., 2010; Berrocal et al., 2013; Liu et al., 2017; Ma et al., 2018, 2020; Burks et al., 2021; Muangsanit et al., 2021) the repair group with implanted SCs demonstrated statistically similar number of myelinated axons as the reversed autograft group that regenerated to the distal segment of the graft used to repair gaps ranging from 10 to 17 mm between 4 and 16 weeks after surgery. Of note, although Aszmann et al. (2008) observed that the number of myelinated axons at the distal segment of a much longer gap, about 30 mm, bridged with a homologous nerve graft that was seeded with harvested SCs were significantly fewer, smaller in diameter and had thinner myelination as compared to the autograft group at 12 weeks after surgery, they performed significantly better than the empty grafts, which did not have any axons reach the distal end.

Retrograde labeling also demonstrated a high level of neuronal regeneration with significant improvement in the number of spinal motoneurons and DRG sensory neurons with the addition of SCs that was not statistically different from the reversed autograft repair (McGrath et al., 2010; Liu et al., 2017; Wang et al., 2017; Huang et al., 2019; Ma et al., 2020). Interestingly, Huang et al. (2019) demonstrated that upregulation of neurotrophic factor expression in tetracycline-responsive transcriptional activator (Tet-On/c-Jun)-transduced SCs improved neuronal regeneration of both sensory and motor neurons with doxycycline treatment in a time-restricted manner as compared to repair with wild type SCs.

Regenerated axon morphology was measured either through axonal area (Berrocal et al., 2013; Gonzalez-Perez et al., 2018) or diameter (Aszmann et al., 2008; Chang, 2009; Liu et al., 2017; Wang et al., 2017; Ma et al., 2018, 2020). As with axon count, the repair of long gaps with grafts supplemented with SCs demonstrated larger areas (Berrocal et al., 2013; Gonzalez-Perez et al., 2018) and diameters (Aszmann et al., 2008; Liu et al., 2017; Wang et al., 2017; Ma et al., 2018, 2020; Burks et al., 2021) as compared to the non-SC groups. A high degree of axon myelination was also observed among the SC groups (Aszmann et al., 2008; Sun et al., 2009; Liu et al., 2017; Wang et al., 2017; Ma et al., 2018, 2020; Burks et al., 2021).

These studies provide evidence that repair of critical nerve gap defects with grafts infused with SCs significantly increases the rate of axonal regeneration and myelination near equal to that of the optimal reversed autograft control.

### Effects on microvasculature

There were two studies that looked at the effects on microvasculature with microvessel density (MVD) measurements within the repair site (Liu et al., 2017; Ma et al., 2018). Ma et al. (2018) observed a significant increase in MVD with SC supplementation. Specifically, they explored the use of PFTBA fibrin hydrogel, as an oxygen carrier, within collagen chitosan conduits to increase oxygenation of SCs. Although the group with the PFTBA hydrogel and SCs had the greatest MVD, SC supplementation without the PFTBA hydrogel had greater MVD than the groups repaired with the standard fibrin hydrogel alone and the PFTBA hydrogel alone. Similarly, in a study by Liu et al. (2017) looking at the effects of a magnetic nanocomposite scaffold (MG) with an applied magnetic field (MF) to supplement long gap nerve repair, groups with implanted SCs demonstrated significantly greater MVD than both non-SC groups, MG alone and MG with MF. However, the group with SCs and an applied magnetic field demonstrated the best MVD. These studies suggest that SCs are able to independently stimulate vascularization, which may be further enhanced by an additional stimulus, such as improvement in oxygenation. Though Hoben et al. did not measure objective outcomes relating to graft vascularity, they demonstrated that acellular nerve grafts supplemented with VEGF alone were inferior to acellular nerve grafts loaded with SCs (Hoben et al., 2015).

### Functional recovery

Motor functional recovery was assessed through neurophysiological studies, specifically through conduction studies and compound muscle action potential (CMAP) waveform analysis. Nearly all studies demonstrated enhanced CMAP amplitudes among the respective group with implanted SCs (Sun et al., 2009; Das et al., 2017; Wang et al., 2017; Ma et al., 2018, 2020; Burks et al., 2021), all measured at varying times that ranged from 4 weeks to 12 months. Conduction velocities and latencies, mostly measured at the gastrocnemius and occasionally at the tibialis anterior, were also observed to significantly improve in the SC groups (Sun et al., 2009; Das et al., 2017; Wang et al., 2017; Ma et al., 2018, 2020; Burks et al., 2021). In Burks et al. (2021), onset latencies at the level of the gastrocnemius were observed to be statistically similar between the SC group, the reversed autograft group and the contralateral uninjured control and all were significantly shorter than the conduit alone.

Sensory recovery was assessed by measuring thermal withdrawal latency (Aszmann et al., 2008; Liu et al., 2017; Wang et al., 2017; Gonzalez-Perez et al., 2018; Ma et al., 2018, 2020). Although good sensory recovery among all groups was observed in most studies, there were a few studies were SC groups demonstrated significantly quicker responses to thermal stimuli than all other groups and were statistically similar to the reversed autograft group (Liu et al., 2017; Ma et al., 2018, 2020). Aszmann et al. (2008) was the only study that observed no motor and poor sensory recovery in all groups.

The extent of muscle recovery following denervation was assessed either through measuring the gastrocnemius muscle fiber areas or with muscle weights. The largest gastrocnemius muscle fiber areas at 12 weeks were observed among the SC groups and were not statistically different from the reversed autograft groups (Liu et al., 2017; Ma et al., 2018, 2020). Similarly, the SC groups were also observed to have significantly greater muscle weight recovery as compared to the non-SC groups in some studies (Wang et al., 2017; Burks et al., 2021). However, in McGrath et al. (2010), best muscle weight recovery at 16 weeks was observed among the autograft group, followed by the BD hydrogel conduits and then the standard alginate/fibronectin hydrogel conduits. The addition of SCs were not observed to impart any statistical improvement in muscle recovery in any of the hydrogel conduits. Interestingly, upregulation of neurotrophic factor expression in Tet-On/c-Jun-transduced SCs in Huang et al. (2019), also significantly increased muscle fiber areas and muscle weight in time-restricted manner as compared to wild type SCs. Lastly, Wang et al. (2017) performed motor end plate analysis on the tibialis anterior muscle and observed a significant increase in the number of motor end plates among the groups with SCs as compared to the non-SC group. These results suggest that SCs may aid in reducing muscle atrophy, possibly by enhancing neuronal regeneration of motor neurons and increasing motor end plates.

Finally, significant improvement in hindlimb functional recovery with SC implantation as measured by sciatic functional index (SFI) scores from walking track analysis was observed in a few studies (Liu et al., 2017; Ma et al., 2018, 2020; Burks et al., 2021; Yao et al., 2021). In contrast, Das et al. (2017) did not observe improvement in SFI scores with SC implantation, and instead observed that improvement was more dependent on the conduit composition.

## Discussion

### Past

Physicians and scientists have long grappled with the challenges of peripheral nerve repair and nerve regeneration. In the fifth century BC, Hippocrates stated in his works, *On the Articulations* and *Aphorisms*, that care should be taken to avoid overstretching nerves, and that cut nerves would neither unite nor be restored (Belen et al., 2009). Further advancements were made by Galen in the second and early third centuries, as he identified that nerves were responsible for varying functions, and that injury or loss resulted in, what was thought to be, permanent insensitivity and paralysis. In the seventh century, Paul of Aegina, a Byzantine Greek physician, first described an attempt at peripheral nerve repair (Paulus., 1528). Experiments done on the glossopharyngeal and hypoglossal nerve of frogs led to the characterization of neuronal degeneration after injury, described by Augustus Waller in the 1850s (Waller, 1851). In the late 1800s, Gluck attempted to use a piece of hollow bone as a nerve conduit to repair peripheral nerve injury. This unsuccessful effort was followed by several experiments by Vanlair, who was able to achieve nerve regeneration using a similar conduit made of bone (Battiston et al., 2005).

The implementation of aseptic surgical technique at the turn of the century made peripheral nerve repair increasingly possible, and the dawn of human genomics has provided researchers with abundant frontiers to explore in this area. In the past several decades, we have experienced the birth and evolution of synthetic conduits for use in peripheral nerve repair, starting as hollow tubes made from silicone and transforming into meticulously engineered structures made from biomaterials such as collagen, chitosan, and cellulose. These conduits have been further augmented in recent years to include neurotrophic factors to aid in SC recruitment, and ultimately loaded with SCs themselves in an effort to improve nerve regeneration.

### Present

Peripheral nerve repair continues to pose unique challenges to surgeons and patients alike. Long considered the gold standard, harvesting the sural nerve for transplantation requires an additional surgery, increasing the chance for iatrogenic complications, post-operative infection, as well as infliction of added sensory deficits along the nerve's distribution. This procedure has the potential to result in neuroma formation leading to neuropathic pain, as well as additional time repositioning and operating. In a study of 478 sural nerve harvest procedures, 92.9% of patients experienced sensory deficits, nearly 19.7% experienced chronic pain post-operatively, and 5.7% were complicated by wound infection at the harvest site (Kawamura et al., 2010; Ducic et al., 2020). Additionally, the ability to repair peripheral nerve injuries is limited by the supply of sural nerve, which may be insufficient, especially in the case of large diameter or long gap injuries. Furthermore, as there are anatomic variations from patient to patient, the cross-sectional area of the sural nerve may not be sufficient to adequately bridge a sciatic nerve transection, for example. A recent meta-analysis on 3,974 limbs revealed a pooled mean length of the sural nerve to be 14.78 (±5.76) cm with a mean diameter of 0.28 (±0.03) cm (Ramakrishnan et al., 2015). The sciatic nerve is known to have a diameter of ~2 cm, roughly 6.5 times greater than the average sural nerve. Therefore, peripheral nerve surgeons have resulted in commonly prioritizing coaptation of the medial fibers of the sciatic nerve in hopes of maximizing motor function over restoration of sensation in the peroneal division (Gousheh et al., 2008; Burks et al., 2014). Taken together, the autograft has long been the gold-standard for peripheral nerve gap repair, but this technique has many drawbacks and alternative strategies are warranted.

Autologous SC loaded nerve conduits have continuously adapted over time, with modifications to their composition as well as their intra-luminal structures. Recent advancements in technology have allowed for the development of nerve conduits made from silicon, collagen, polycaprolactone (PCL), chitosan, and various other synthetic polymers to bridge peripheral nerve gaps and provide a scaffold to guide nerve regeneration. From 1995 to 2012, 11 neural regeneration guide devices were approved for use by the FDA (Kehoe et al., 2012). Hollow synthetic tubes were the first generation of conduits employed, but use of these tubes were largely limited to small-gap injuries and improvements in design were needed to target longer gaps (De Ruiter et al., 2009).

Recently, second-generation and tissue-engineered axon guidance channels were developed from biomaterials with the goal of more closely mimicking natural perineurium anatomy, providing increased porosity and optimized architecture within the larger bore tube to aid in the longitudinal growth of regenerating axons (Gaudin et al., 2016; Stewart et al., 2020). Coupled with the structural advancements made, strides have also been made in optimizing the cocktail and concentration of neurotrophic factors, cell types, as well as exosomes and nanoparticles that conduits may be supplemented with. Though commercialization of nerve conduits has allowed for increased availability to surgeons, autografts and allografts continue to outperform conduits in their current forms (Mauch et al., 2019; Herman and Ilyas, 2020). However, the supplementation of Schwann cells within the conduits offers promise in improving outcomes.

Taken together, the results of our systematic review provide strong evidence that conduits loaded with autologous SCs are significantly superior to conduits alone in improving axonal regeneration and functional outcomes after long-gap peripheral nerve injury. Additionally, conduits loaded with SCs performed statistically similar to reverse autografts in the seven studies in which positive controls were implemented.

Berrocal et al. (2013) demonstrated at our own institution that conduits or processed nerve allografts loaded with autologous SCs enhanced regeneration of myelinated axons as compared to an acellular tube. With these promising results in mind, the first in-human autologous SC use was performed at our institution in 2015 (Levi et al., 2016). A sural nerve biopsy and tissue sample from traumatized sciatic nerve stumps was taken in order to expand a population of autologous SCs. Sural nerve grafts were combined with an FDA-approved collagen matrix, which was seeded with the autologous human SCs, bridging a 7.5 cm gap. The patient demonstrated improvement in plantar flexion, from 0/5 to 4/5, at 18 months after surgery. The safety and feasibility of this procedure was corroborated by a second case at our institution in which a gunshot wound that resulted in a 5 cm partial disruption of the tibial division of the sciatic nerve was repaired in a similar manner. The patient regained all motor function associated with the tibial nerve and partial sensation (Gersey et al., 2017). More recently, the first phase 1 clinical trial for transplantation of autologous SCs in chronic spinal cord injury demonstrated that transplants were well-tolerated with no significant adverse events occurring (Gant et al., 2022).

Though these clinical outcomes are extremely promising, widespread implementation of this technology would not be feasible without a standardized and reproducible protocol for SC isolation and expansion in culture. Our group has published extensively on the investigation and refinement of protocols for successful SC harvesting and propagation in animal and, more recently, human subjects. Once it had been established that adult rat SCs could be cultured whilst maintaining their ability to myelinate neurons, the process of optimizing the right combination of neural growth factors in cell culture began (Morrissey et al., 1991). Shortly thereafter, the initial work on harvesting and expanding human SCs using the proper mitogens took place (Levi and Bunge, 1994; Casella et al., 1996). These human SCs were successfully transplanted into the peripheral nerves of immune deficient mice, however these grafts did not function as well in bridging an 8 mm nerve defect as grafts containing autologous murine SCs (Levi et al., 1994). This experimentation established NGF, BDNF, CNF, and rHRG, best in combination with cAMP, as crucial neurotrophic factors and potent mitogens in the promotion of neural regeneration (Levi et al., 1995). Additionally, the number of cell-culture passages in order to obtain high SC purity was reduced (Levi, 1996). Then, in 1997, a study at our institution was undertaken to repair a 15 mm nerve gap in primates using autologous SCs in a nerve conduit. These results demonstrated that, although the sural autograft group was superior, all groups regained some degree of functionality with the sural autograft and SC infused conduit groups performing better in electrophysiological outcomes (Levi et al., 1997).

More recently, we published our optimized protocol for human autologous SC isolation, purification, and expansion, in which the average yield per patient was 87.2 ± 89.2 million cells at P2 and 150.9 ± 129.9 million cells at P3 with over 90% purity and viability (Khan et al., 2022). Utilizing this protocol, investigators can reliably harvest human SCs for use in future human clinical trials. In the setting of peripheral nerve injury, SCs may be harvested from the injury site itself and expanded in culture while allowing 3–4 weeks for nerve end maturation. This period allows sufficient time for purification and expansion in culture with the added benefit of allowing the surgeon to clearly make out fascicular anatomy at the time of surgery.

Further studies are needed to fully explore whether conduits facilitating oxygen perfusion, magnetic scaffolds using nanoparticles, or scaffolds lined with other cell types such as endothelial cells confer translational advantages in peripheral nerve repair.

### Future

Several paradigms remain controversial in peripheral nerve regeneration studies. Though many conduits are currently available and novel conduits continue to emerge, there has not been a definitive consensus established across the literature as to which conduit is superior (Arslantunali et al., 2014; Pabari et al., 2014; Muheremu and Ao, 2015). Contemporary conduits have been engineered to more closely mimic neuronal structure, with longitudinal tracts for cells to orient themselves across. In studies that have successfully loaded SCs into nerve conduits, there are often challenges with maintaining SC viability, especially as conduits become longer to bridge large nerve gaps. Therefore, several studies have been conducted in which nerve conduits are augmented with novel technologies aimed at increasing vascular perfusion, increasing SC stimulation, and facilitating increased SC survival. Studies reviewed herein employed conduits which were augmented *via* the use of BD™ PuraMatrix™ peptides, crosslinked with genipin, hydrogels made of collagen, fibronectin, or neurotrophic factors, as well as PFTBA to augment oxygen delivery (Chang, 2009; McGrath et al., 2010; Gonzalez-Perez et al., 2018; Ma et al., 2018, 2020). Implementation of magnetic scaffolds and longitudinally oriented magnetic fields has also been utilized to augment nerve regeneration (Gordon, 2016; Liu et al., 2017). Other conduits have been engineered from biomaterials to aide in biocompatibility and biodegradation (Fornasari et al., 2020).

Objective differences in the regeneration of sensory vs. motor nerve fibers is an area of continued investigation. Several studies have demonstrated similar outcomes in sensory and motor nerve regeneration speed and outcomes (Moldovan et al., 2006; Ali et al., 2019). However, other studies suggest the architectural differences between motor and sensory nerves cause different outcomes to be achieved (Moradzadeh et al., 2008; Jianping et al., 2012). It has also been suggested that motor or mixed motor-sensory nerve grafts perform superior to sensory nerve grafts in autologous nerve grafting, calling into question whether there exist architectural differences facilitating this superior effect, or differing expression profiles in the SCs corresponding to each of these graft types (Nichols et al., 2004).

On this note, SCs harvested from different nerves may have differing effects on axon regeneration, as their expression profiles may vary. For example, after nerve transection, the proximal stumps of injured peripheral nerves have been shown to down-regulate genes involved in myelination and upregulate genes associated with growth and repair, encouraging the “repair” SC phenotype (Jessen and Mirsky, 2016, 2019). Distal nerve stumps have been noted to upregulate genes associated with clearing cellular debris, making way for the regenerating fibers (Menorca et al., 2013). As each donor site may exhibit a different predominant SC phenotype, the optimal site for SC harvesting, be it the proximal stump of the patient's injured nerve, the distal stump, or a sensory nerve has yet to be determined. As was investigated by Huang et al. (2019), whether SCs can be harvested and pushed to express genes encouraging neuronal regeneration prior to transplantation remains to be seen. Putting pro-regenerative genes such as c-Jun under transcriptional control of drugs would effectively allow clinicians to manipulate SC phenotypes post-autologous transplantation based on clinical information. As the expression profiles of SCs in the setting of neurotmesis are characterized in increasing detail, studies aiming to transfect and overexpress certain neurotrophic genes within transplanted SCs may provide additional benefits in future studies, allowing clinicians to change expression profiles as appropriate, pushing for regenerative phenotypes in early stages of healing, and for myelination phenotypes after axons have bridged the gap.

One way to achieve the pro-regenerative signal conveyed by SCs and forgo the tedious process of SC harvesting and expansion for transplantation is the use of exosomes. This technology is currently under investigation as a potential alternative or augmentation to autologous SC therapy (Ching and Kingham, 2015; Hessvik and Llorente, 2018; Qing et al., 2018). To collect exosomes, SCs are isolated and observed to express pro-regenerative genes. The ultrafiltrate, containing exosomes, is isolated from the cells and can be loaded into nerve conduits in lieu of SCs or in combination with SCs for use in peripheral nerve repair. Studies are warranted in which conduits loaded with autologous SCs are compared directly to conduits loaded with pro-regenerative exosomes. Having a large supply of exosomes readily available would potentially circumvent the arduous and limiting process of SC harvesting from each patient, overcome the need for immunosuppressive agents, and allow for widespread implementation in the clinic. Therefore, SC exosomes in combination with conduits offer great translational potential and have yet to be fully investigated. We look forward to continued utilization of conduits loaded with autologous SCs for peripheral nerve repair at our institution and others as well as continued studies aimed at optimization of this technology *via* the use of exosomes in future investigations.

### Limitations

The 17 studies included in our review investigate varying outcomes relating to SC transplantation, making direct comparisons between studies difficult. Additionally, methodology pertaining to conduits used, number of cells transplanted, and controls implemented varied greatly between studies, which may affect interpretations drawn from pooling the studies together for analysis. All included studies were undertaken using mice or rats as models. These models have been posited to be poor models for human peripheral nerve injury due to their small size, species specific neurobiological regenerative profile, and unreliable extrapolation to humans (Kaplan et al., 2015). Individual studies were not weighted in any way prior to analysis. One obvious limitation is the paucity of clinical data available regarding autologous SC transplantation in peripheral nerve injury repair. Lastly, further characterization of harvesting, expansion, and transplantation protocol regarding SCs would greatly improve the external validity of the results reviewed.

## Conclusion

Here, we review 17 *in-vivo* studies on autologous SC transplantation for use in long-gap, ≥10 mm, peripheral nerve gap repair. This study provides evidence that nerve conduits loaded with autologous SCs are superior to conduits alone. Furthermore, conduits loaded with autologous SCs perform similarly to optimal reversed-autograft controls both by histo-morphometric parameters as well as functional outcomes. Further studies are needed to establish optimal conduit composition, loaded SC cell density, and additional technologies that can be used to augment the regenerative capabilities of SCs in long-gap peripheral nerve injuries.

## Data availability statement

The original contributions presented in the study are included in the article/supplementary material, further inquiries can be directed to the corresponding author.

## Author contributions

FV, AD, EE, TS, AK, RS, and AB conducted literature search, drafted, and edited manuscript. Y-SL, SB, and AL directed the project, formulated study idea, and assisted in editing and revising manuscript. All authors contributed to the article and approved the submitted version.

## Conflict of interest

The authors declare that the research was conducted in the absence of any commercial or financial relationships that could be construed as a potential conflict of interest.

## Publisher's note

All claims expressed in this article are solely those of the authors and do not necessarily represent those of their affiliated organizations, or those of the publisher, the editors and the reviewers. Any product that may be evaluated in this article, or claim that may be made by its manufacturer, is not guaranteed or endorsed by the publisher.

## Abbreviations

SC: Schwann Cell
SCLC: Schwann Cell-like cells.
